# A single dose of a vesicular stomatitis virus-based influenza vaccine confers rapid protection against H5 viruses from different clades

**DOI:** 10.1038/s41541-019-0155-z

**Published:** 2020-01-10

**Authors:** Wakako Furuyama, Pierce Reynolds, Elaine Haddock, Kimberly Meade-White, Mai Quynh Le, Yoshihiro Kawaoka, Heinz Feldmann, Andrea Marzi

**Affiliations:** 1 Laboratory of Virology, Division of Intramural Research, National Institute of Allergy and Infectious Diseases, National Institutes of Health, Hamilton, MT USA; 2 National Institute of Hygiene and Epidemiology, Hanoi, Vietnam; 3 Department of Pathobiological Sciences, University of Wisconsin-Madison, Madison, WI USA; 4 Division of Virology, Department of Microbiology and Immunology, Institute of Medical Science, Univeristy of Tokyo, Tokyo, Japan; 5 Present Address: Mayo Clinic Graduate School of Biomedical Sciences, Rochester, MN USA

**Keywords:** Live attenuated vaccines, Influenza virus

## Abstract

The avian influenza virus outbreak in 1997 highlighted the potential of the highly pathogenic H5N1 virus to cause severe disease in humans. Therefore, effective vaccines against H5N1 viruses are needed to counter the potential threat of a global pandemic. We have previously developed a fast-acting and efficacious vaccine against Ebola virus (EBOV) using the vesicular stomatitis virus (VSV) platform. In this study, we generated recombinant VSV-based H5N1 influenza virus vectors to demonstrate the feasibility of this platform for a fast-acting pan-H5 influenza virus vaccine. We chose multiple approaches regarding antigen design and genome location to define a more optimized vaccine approach. After the VSV-based H5N1 influenza virus constructs were recovered and characterized in vitro, mice were vaccinated by a single dose or prime/boost regimen followed by challenge with a lethal dose of the homologous H5 clade 1 virus. We found that a single dose of VSV vectors expressing full-length hemagglutinin (HAfl) were sufficient to provide 100% protection. The vaccine vectors were fast-acting as demonstrated by uniform protection when administered 3 days prior to lethal challenge. Moreover, single vaccination induced cross-protective H5-specific antibodies and protected mice against lethal challenge with various H5 clade 2 viruses, highlighting the potential of the VSV-based HAfl as a pan-H5 influenza virus emergency vaccine.

## Introduction

Influenza A viruses, which belong to the family *Orthomyxoviridae*, have a single-stranded negative-sense RNA genome consisting of eight segments.^1^ They are important zoonotic pathogens, with high morbidity in pigs, horses, poultry, and humans.^2^ Influenza A viruses have two envelope glycoproteins (GPs), hemagglutinin (HA) and neuraminidase (NA), and are divided into subtypes based on antigenicity. Subtypes H1-16 HA and N1-9 NA have been isolated from water birds, the natural reservoir of influenza A viruses.^3,4^ Until 1997, avian influenza A viruses were considered unlikely to be transmitted directly to humans because they do not bind the human sialic acid-α2,6-galactose (SAα2,6Gal) receptor with high affinity.^5^ However, highly pathogenic avian influenza (HPAI) viruses can be transmitted from wild birds upon close contact causing sporadic outbreaks in domestic poultry. This happened for the first time in 1997 in Hong Kong when 18 human cases of respiratory illness, including six fatalities, were caused by HPAI subtype H5N1 viruses.^6–8^ Since then, 860 human cases, with 454 deaths (~53% case fatality rate), have been reported by the World Health Organization.^9^ Furthermore, some reassortant H5 viruses with different NA subtypes (e.g. H5N2, H5N8, and H5N6) originated from the same ancestral H5N1 virus, and have recently emerged in China and spread to other countries in Eurasia and North America.^10–15^ Since some HPAI viruses are resistant to the currently available treatment options for influenza A virus infections namely oseltamivir, amantadine, and interferon (IFN),^16,17^ the development of vaccines is an ongoing effort of high priority for public health to be prepared for a potential epidemic or pandemic of HPAI.

Several different vaccination strategies have been developed against influenza A viruses including inactivated whole virus, live-attenuated influenza virus, viral vectors, and DNA vaccines.^18^ Currently, the FDA-approved and licensed whole virus and live-attenuated vaccines against human influenza A viruses are mainly produced in embryonated chicken eggs and the manufacturing process can take up to 9 months.^18–20^ Unfortunately, the high pathogenicity of HPAI viruses for the chicken embryo reduces virus growth complicating efforts to obtain quality allantoic fluid with high virus titers. Therefore, HPAI viruses are not suitable as seed viruses for inactivated virus-based vaccine production.

Vesicular stomatitis virus (VSV) is a single-stranded negative-sense RNA virus in the family *Rhabdoviridae*. Although VSV can cause disease in livestock and other animals, it is highly restricted by the human IFN response and generally does not cause any or only very mild disease.^21^ The VSV platform used here is based on the attenuated replication-competent vaccine that produces a rapid and robust immune response to foreign antigens after a single immunization and has been shown to protect against numerous pathogens.^22–26^ Especially, the VSV-based Ebola virus (EBOV) vaccine, VSV-EBOV (also known as rVSV-ZEBOV or Ervebo), which expresses the EBOV GP instead of the VSV GP, is considered safe and highly immunogenic based on data from multiple clinical trials.^27,28^ Noteworthy, VSV-EBOV has shown promising efficacy against EBOV in a phase III clinical trial^28^ and is currently being used in the Democratic Republic of the Congo during the ongoing EBOV outbreak.^29^ The promising safety profile of this live-attenuated vaccine and the favorable immune cell targeting mediated by the EBOV GP makes VSV-EBOV an interesting platform for vaccine development.^30^ The feasibility of this concept has previously been demonstrated in preclinical studies with vaccines for influenza (HPAI virus), flavi- (Zika virus), and bunyaviruses (Andes virus).^24,31–33^

In this study, we designed and tested different VSV-EBOV-based vaccine vectors expressing different versions of the H5N1 HA (A/Vietnam/1203/2004 (VN/1203)) to demonstrate the feasibility of the platform for a fast-acting pan-H5 vaccine. Mice were vaccinated with a single dose or prime/boost regimen of the different vaccine candidates and challenged with a lethal dose of homologous H5N1 virus. We found that a single vaccination with VSV-vectors expressing the full-length HA (HAfl) induced cross-reactive H5-specific antibodies and conferred complete protection against lethal challenge with various H5 clade 2 viruses. Furthermore, a single dose of these vaccine vectors provided uniform protection in mice against lethal H5N1 challenge within 3 days after vaccination.

## Results

### Construction and characterization of VSV vaccine vectors

**Fig. 1:**
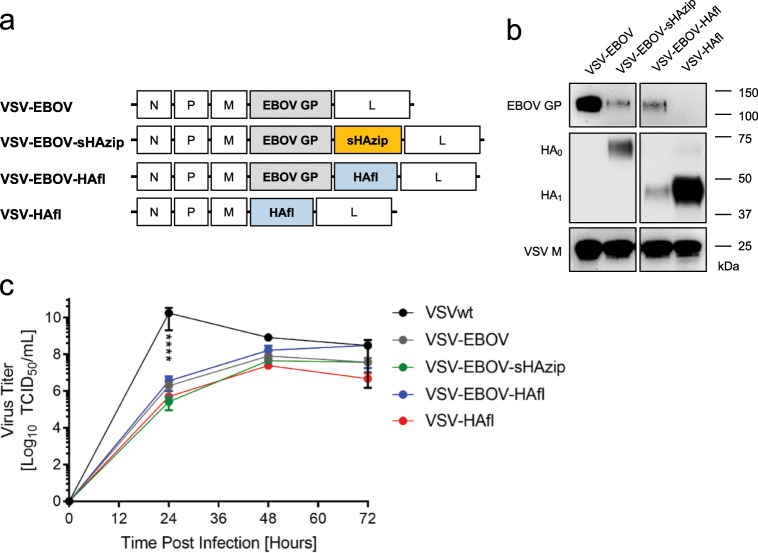
Design and in vitro characterization of VSV vectors. **a** Schematic representation of VSV-HA vectors. N nucleoprotein, P phosphoprotein, M matrix protein, EBOV GP Ebola virus glycoprotein, L polymerase, sHAzip soluble HA protein with trimerization sequence, HAfl full-length HA protein. **b** Western Blot analysis confirmed the presence of HA, EBOV GP, and VSV M in the supernatant of infected Vero E6 cells. Relevant parts of the gels are shown. For uncropped gel images, see Supplementary Fig. 1. **c** Growth kinetics of VSVs propagated on Vero E6 cells. The mean and standard deviation of one experiment performed in triplicates are shown. Statistically significant differences are indicated (*p* < 0.0001 (****)).

We generated VSV-based H5 vaccine vectors by inserting either the full-length open reading frame (ORF) of the H5N1 HAfl (VN/1203) or a soluble version of this gene lacking the transmembrane and cytoplasmic domains but carrying a mutated single-basic cleavage site to prevent cleavage in the cells and a GCN4 leucine zipper domain (sHAzip) for stabilization of the trimeric structure into the VSV-EBOV vector^34,35^ (Fig. 1a). This sHAzip antigen has previously been shown to be protective in chickens as a subunit vaccine.^34^ We also generated a VSV vector expressing the H5N1 HAfl alone without the EBOV GP (VSV-HAfl; Fig. 1a)^36^ in order to control for the contribution of the EBOV GP to vaccine efficacy. Expression of the different H5 antigens from the VSV vectors was confirmed by subjecting the supernatant of infected cells to SDS-PAGE and immunoblotting (Fig. 1b, Supplementary Fig. 1). First, we showed the presence of VSV particles by detecting the VSV matrix (M) protein in the supernatant of infected cells (Fig. 1b, Supplementary Fig. 1). The incorporation of EBOV GP into VSV particles differed among the vectors and was, as expected, highest for VSV-EBOV for which it is the only surface protein and antigen encoded by this vector (Fig. 1b, Supplementary Fig. 1). Expression of sHAzip was verified by detecting the non-cleaved sHA_0_ precursor likely secreted from infected cells. HAfl expression was demonstrated by detecting the furin-cleaved fragment HA_1_ in mature spikes on VSV particles. As expected, the incorporation of HAfl into recombinant VSV particles was much stronger for VSV-HAfl compared to VSV-EBOV-HAfl, likely because it is the only surface GP and encoded antigen in the VSV vector.

Next, we performed a series of studies measuring the rate and extent of vaccine virus growth over time. Vero E6 cells were infected in triplicate with each VSV-based vector (multiplicity of infection (MOI) 0.01) and samples were collected from the supernatant at 24, 48 and 72 h for titration. Wild-type VSV (VSVwt) grew more rapidly and to significantly higher titers early post infection compared to any of the other recombinant VSV-based vectors (Fig. 1c). We did not observe any significant difference in the growth kinetics of VSV-based vectors expressing either one (VSV-EBOV, VSV-HAfl) or two foreign antigens (VSV-EBOV-sHAzip, VSV-EBOV-HAfl) with most vectors reaching peak titers between 10^7^ and 10^8^ TCID_50_/ml at 48 h suggesting that the expression of a second antigen did not significantly further attenuate VSV-EBOV (Fig. 1c).

### Efficacy of the VSV vaccine vectors against lethal HPAI challenge in mice

**Fig. 2:**
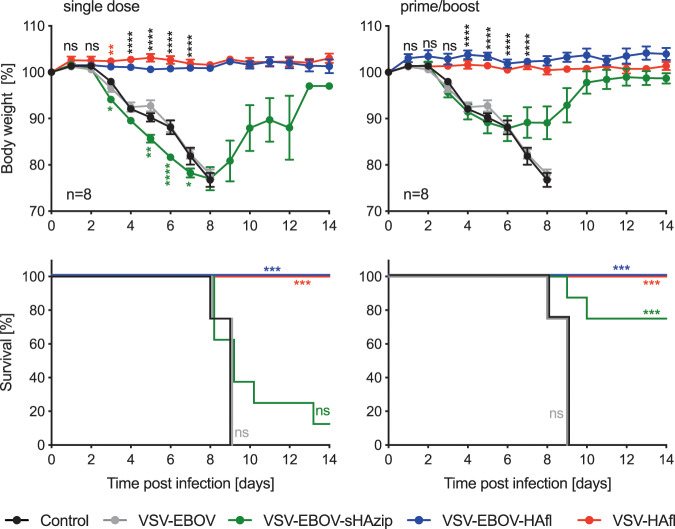
Efficacy of the VSV-EBOV-HA influenza virus vectors against lethal HPAI H5N1 influenza virus challenge. Groups of eight mice were IM vaccinated with a single dose (left) or a prime/boost (right) of the indicated VSVs on day −21 (left, single dose), or on days −42 and −21 (right, prime/boost) before challenge. On day 0, mice were challenged IN with a lethal dose (400 TCID_50_) of HPAI H5N1 virus. The animals were monitored for clinical signs of infection and body weight was recorded daily. Body weight (upper panels) and survival curves (lower panels) are shown. Error bars indicate standard error of the mean (SEM). Statistically significant differences are indicated in colors corresponding to the vaccine group (*p* < 0.0001 (****), *p* < 0.001 (***), *p* < 0.01 (**), *p* < 0.05 (*); ns, not significant).

Groups of 16 female Balb/c mice were intramuscularly (IM) vaccinated with 1 × 10^4^ plaque forming units (pfu) of VSV-EBOV, VSV-EBOV-sHAzip, VSV-EBOV-HAfl, or VSV-HAfl 21 days (single-dose vaccination) or 42 and 21 days (prime/boost-vaccination) prior to challenge. On day 0, the mice were intranasally (IN) challenged with 100 LD_50_ (400 TCID_50_) of HPAI H5N1. As expected, control and VSV-EBOV vaccinated mice succumbed to the lethal H5N1 challenge within 9 days (Fig. 2). Single-dose vaccination of the VSV-EBOV-sHAzip showed only 12.5% protection against H5N1 infection with severe weight loss (Fig. 2, left panels). Prime/boost-vaccination improved the outcome of the VSV-EBOV-sHAzip resulting in mild disease as evidenced by temporary weight loss and moderate disease with 75% survival for VSV-EBOV-sHAzip (Fig. 2, right panels). In contrast, single and prime/boost vaccination with VSV-EBOV-HAfl or VSV-HAfl protected 100% of the mice from lethal challenge with no signs of clinical disease (Fig. 2).

In order to improve the protective efficacy of the VSV-sHAzip vector, we wanted to increase the antigen expression levels. Therefore, the sHAzip or sHA (without trimerization domain) antigens were inserted further upstream into the VSV-EBOV backbone resulting in two additional vaccines, VSV-sHAzip-EBOV and VSV-sHA-EBOV (Supplementary Fig. 2A). These vaccine viruses were recovered and antigen expression was confirmed in the cell supernatant similarly to the previously generated vaccines (Supplementary Fig. 1). In vitro growth kinetics demonstrated no difference in comparison to the other VSV constructs (Fig. 1b, Supplementary Fig. 2B). Next, we analyzed the protective efficacy of these improved VSV-sHA-EBOV vaccine candidates in mice. Single-dose and prime/boost-vaccinations with the VSV-sHAzip-EBOV revealed similar protective efficacies compared to VSV-EBOV-sHAzip (Fig. 2, Supplementary Fig. 2C). Interestingly, the H5N1 challenge of VSV-sHA-EBOV-vaccinated mice demonstrated higher survival rates compared to sHAzip expressing VSV vectors (Fig. 2, Supplementary Fig. 1D).

Taken together, the challenge experiments demonstrated that HAfl is the superior antigen to any of the sHA versions as a single dose results in uniform protection using the VSV platform. Interestingly, sHA performed better than sHAzip.

### Analysis of VSV-based vaccine-mediated antibody responses

**Fig. 3:**
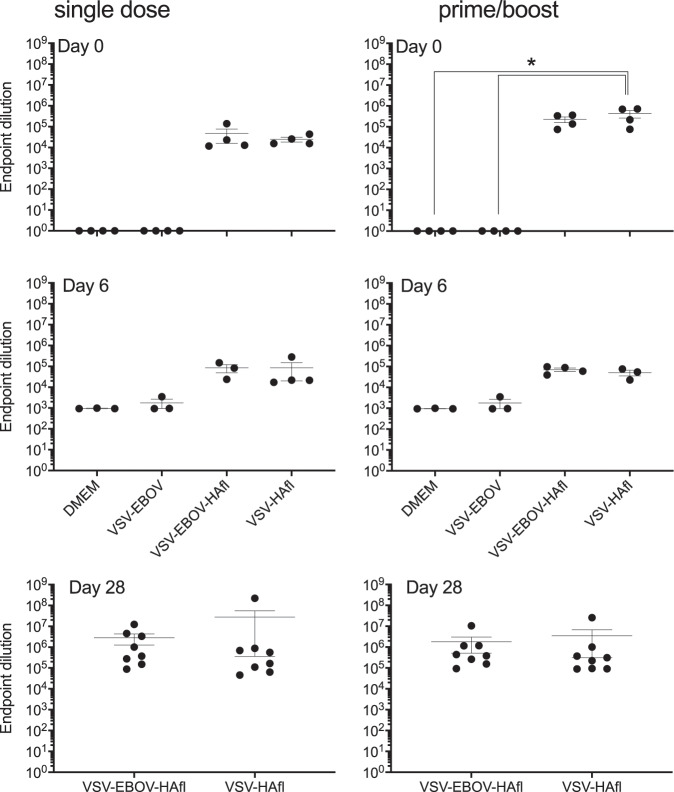
Antibody response after vaccination and challenge. Levels of HA (H5)-specific IgG in the mouse serum samples were analyzed by ELISA. Serum samples from the single dose or prime/boost vaccination with the HAfl-expressing VSVs were collected on days 0 (*n* = 4), 6 (*n* = 3 or 4), and 28 (survivors, *n* = 8) post challenge. Error bars indicate standard deviation. Statistically significant differences are indicated (*p < 0.05).

Total anti-HA (H5) immunoglobulin G (IgG) and neutralizing antibody responses from all VSV-vaccinated mice were analyzed in serum samples collected directly prior to challenge (day 0) and day 6 and day 28 post challenge. Enzyme-linked immunosorbent assay (ELISA) was performed to determine total anti-HA IgG levels in the serum of the mice over time (Fig. 3, Supplementary Fig. 3). In control and VSV-EBOV-vaccinated mice, we observed no antibody responses on day 0, but HA-specific IgG was detected on day 6 after H5N1 challenge with all animals succumbing to infection by day 9 (Fig. 3, Supplementary Fig. 3). All HAfl-vaccinated mice responded with HA-specific antibody responses to a single dose on day 0 (Fig. 3, top left panel) that were lower compared to those of the corresponding prime/boost vaccinated mice (Fig. 3, top right panel). The same was observed for sHA/sHAzip-vaccinated mice (Supplementary Fig. 3). VSV-HAfl prime/boost vaccination elicited the highest HA-specific IgG responses that were significantly higher compared to control mice and the mice vaccinated with VSV-EBOV, VSV-sHAzip-EBOV, and VSV-EBOV-sHAzip. In all vaccinated and surviving mice, the H5N1 challenge served as a boost as documented by the increase in HA-specific IgG titers measured on day 28 post challenge (Fig. 3, Supplementary Fig. 3).

### Time to immunity of the VSV-based HAfl vaccines

**Fig. 4:**
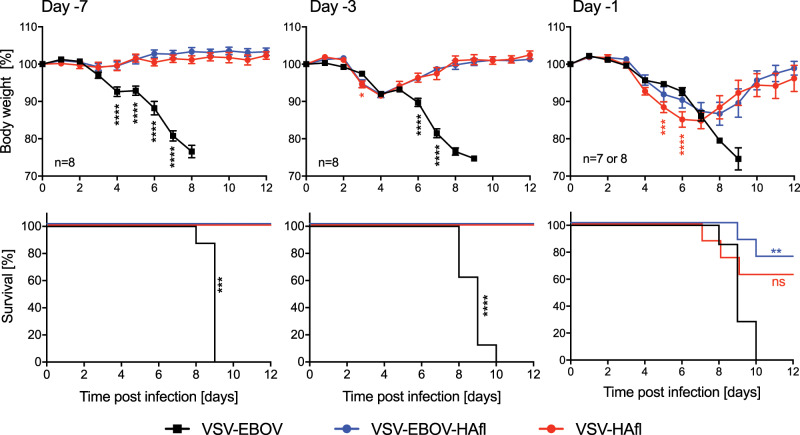
Time to immunity of the VSV-based-HAfl vaccines against lethal dose of H5N1 challenge. Groups of seven or eight mice, respectively, were IM vaccinated with a single dose of VSV-EBOV-HAfl, VSV-HAfl or VSV-EBOV on day 7, 3, or 1 prior to H5N1 challenge. The VSV-EBOV day −1 group only had seven animals. Body weight changes (upper panels) and survival curves (lower panels) after lethal H5N1 challenge are shown. Error bars indicate standard error of the mean (SEM). Statistically significant differences are indicated in colors corresponding to the vaccine group (*p* < 0.0001 (****), *p* < 0.001 (***), *p* < 0.01 (**), *p* < 0.05 (*); ns, not significant).

Finally, we determined the minimum time to immunity for the two most promising vaccine candidates, VSV-EBOV-HAfl and VSV-HAfl. Groups of eight female Balb/c mice were IM vaccinated with 1 × 10^4^ pfu of VSV-EBOV-HAfl or VSV-HAfl on days 7, 3, or 1 prior to lethal homologous H5N1 challenge. We found that both vaccines resulted in 100% protection with no or little weight loss when mice were vaccinated at least 3 days prior to challenge, whereas VSV-EBOV-vaccinated mice succumbed to infection within 10 days (Fig. 4). Furthermore, the day −1 vaccinations resulted in partial survival with 62.5% for VSV-HAfl and 75% for VSV-EBOV-HAfl (Fig. 4). Overall, the data demonstrate that both vaccine candidates are equally potent inducers of rapid protection with a slight but not statistically significant benefit of VSV-EBOV-HAfl over VSV-HAfl.

### Cross-protection with a single dose of the VSV-based HAfl vaccines

Due to frequently occurring antigenic changes with influenza viruses, it is important to determine if vaccine candidates elicit antibodies against viruses from different antigenic clades within the same subtype. Therefore, we performed hemagglutinin inhibition (HI) tests to examine the ability of the VSV-based H5N1 vaccines to generate cross-neutralizing antibody responses against heterologous H5 influenza viruses. For this, we used the day 0 mouse serum samples and a panel of nine attenuated candidate vaccine influenza viruses encoding HAs belonging to different H5 clades that were isolated from geographically distinct locations (Supplementary Table 1). We found that prime/boost vaccination with VSV-EBOV-HAfl or VSV-HAfl elicited cross-neutralizing antibodies against all tested clades (Table 1). Cross-neutralizing antibodies were also detected in the single-dose vaccination group of these two vaccines; however, and similar to the total HA-specific IgG, levels were lower and cross-neutralization was not detected for all clades (Table 1). In contrast, all other vaccines expressing the sHA/sHAzip antigen revealed no cross-neutralizing activities after administration of a single-dose and limited cross-neutralizing activity after the prime/boost. These results demonstrated that the VSV-based vaccines expressing HAfl induce more potent cross-neutralizing antibodies than the sHA/sHAzip antigens.

**Table 1:** Cross-protective potential of the antibody response induced by the VSV-EBOV-based HA vaccines in vitro.

Vaccines	Single dose vaccination									
	VN/1203		IBCDC-RG2		IBCDC-RG7		IDCDC-RG29		IDCDC-RG30	
	Reactive	Max titer	Reactive	Max titer	Reactive	Max titer	Reactive	Max titer	Reactive	Max titer
Control	0/4	<10	0/4	<10	0/4	<10	0/4	<10	0/4	<10
VSV-EBOV	0/4	<10	0/4	<10	0/4	<10	0/4	<10	0/4	<10
VSV-EBOV-sHAzip	0/4	<10	0/4	<10	0/4	<10	0/4	<10	0/4	<10
VSV-sHAzip-EBOV	0/4	<10	0/4	<10	0/4	<10	0/4	<10	0/4	<10
VSV-sHA-EBOV	0/4	<10	0/4	<10	0/4	<10	0/4	<10	0/4	<10
VSV-EBOV-HAfl	2/4	1:10	1/4	1:10	1/4	1:10	1/4	1:10	0/4	<10
VSV-HAfl	3/4	1:10	2/4	1:10	2/4	1:10	1/4	1:10	0/4	<10
Vaccines	IDCDC-RG34B		IDCDC-RG35		IDCDC-RG36		IDCDC-RG42A		IDCDC-RG43A	
	Reactive	Max titer	Reactive	Max titer	Reactive	Max titer	Reactive	Max titer	Reactive	Max titer
Control	0/4	<10	0/4	<10	0/4	<10	0/4	<10	0/4	<10
VSV-EBOV	0/4	<10	0/4	<10	0/4	<10	0/4	<10	0/4	<10
VSV-EBOV-sHAzip	0/4	<10	0/4	<10	0/4	<10	0/4	<10	0/4	<10
VSV-sHAzip-EBOV	0/4	<10	0/4	<10	0/4	<10	0/4	<10	0/4	<10
VSV-sHA-EBOV	0/4	<10	0/4	<10	0/4	<10	0/4	<10	0/4	<10
VSV-EBOV-HAfl	1/4	1:10	1/4	1:10	1/4	1:10	1/4	1:10	0/4	<10
VSV-HAfl	2/4	1:10	1/4	1:10	1/4	1:10	1/4	1:10	0/4	<10
Vaccines	Prime/boost vaccination									
	VN/1203		IBCDC-RG2		IBCDC-RG7		IDCDC-RG29		IDCDC-RG30	
	Reactive	Max titer	Reactive	Max titer	Reactive	Max titer	Reactive	Max titer	Reactive	Max titer
Control	0/4	<10	0/4	<10	0/4	<10	0/4	<10	0/4	<10
VSV-EBOV	0/4	<10	0/4	<10	0/4	<10	0/4	<10	0/4	<10
VSV-EBOV-sHAzip	0/4	<10	0/4	<10	0/4	<10	0/4	<10	0/4	<10
VSV-sHAzip-EBOV	1/4	1:10	1/4	1:10	1/4	1:10	1/4	1:10	1/4	1:10
VSV-sHA-EBOV	1/4	1:40	0/4	<10	1/4	1:40	1/4	1:160	1/4	1:40
VSV-EBOV-HAfl	4/4	1:160	4/4	1:40	3/4	1:40	1/4	1:40	4/4	1:40
VSV-HAfl	4/4	1:160	4/4	1:160	4/4	1:160	3/4	1:160	4/4	1:40
Vaccines	IDCDC-RG34B		IDCDC-RG35		IDCDC-RG36		IDCDC-RG42A		IDCDC-RG43A	
	Reactive	Max titer	Reactive	Max titer	Reactive	Max titer	Reactive	Max titer	Reactive	Max titer
Control	0/4	<10	0/4	<10	0/4	<10	0/4	<10	0/4	<10
VSV-EBOV	0/4	<10	0/4	<10	0/4	<10	0/4	<10	0/4	<10
VSV-EBOV-sHAzip	0/4	<10	0/4	<10	0/4	<10	0/4	<10	0/4	<10
VSV-sHAzip-EBOV	1/4	1:10	1/4	1:10	1/4	1:10	0/4	<10	0/4	<10
VSV-sHA-EBOV	0/4	<10	0/4	<10	0/4	<10	1/4	1:10	0/4	<10
VSV-EBOV-HAfl	4/4	1:40	4/4	1:40	4/4	1:10	3/4	1:40	4/4	1:40
VSV-HAfl	4/4	1:160	4/4	1:40	4/4	1:40	4/4	1:40	4/4	1:40

**Fig. 5:**
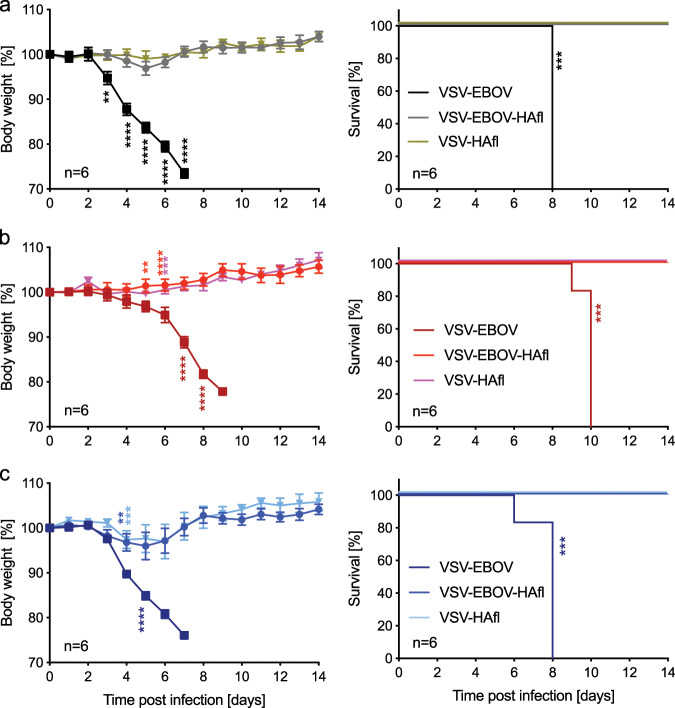
H5 cross-protection after a single vaccine dose. Groups of six mice were IM vaccinated with a single dose of VSV-EBOV-HAfl, VSV-HAfl, or VSV-EBOV on day 28 prior to challenge with 400 TCID_50_ of H5N1 from different clades. **a** A/Anhui/2005 (clade 2.3.4). **b** A/Duck/Vietnam/2010 (clade 2.3.2.1a). **c** A/Indonesia/5/2005 (clade 2.1.3.2). Body weight changes (left panels) and survival curves (right panels) after lethal challenge are shown. Error bars indicate standard error of the mean (SEM). Statistically significant differences are indicated in colors corresponding to the vaccine group (*p* < 0.0001 (****), *p* < 0.001 (***), *p* < 0.01 (**), *p* < 0.05 (*)).

In order to support the cross-protective potential of the VSV-based HAfl vaccines, we vaccinated groups of mice with a single dose of 1 × 10^4^ pfu of VSV-EBOV, VSV-EBOV-HAfl, or VSV-HAfl prior to challenge with a lethal dose of either H5N1 A/Anhui/2005 (clade 2.3.4), H5N1 A/Duck/Vietnam/2010 (clade 2.3.2.1a), or H5N1 A/Indonesia/5/2005 (clade 2.1.3.2). We used 400 TCID_50_, the same challenge dose used with H5N1 A/Vietnam/1203/2004 in the above described experiments. All the VSV-EBOV-vaccinated mice succumbed to infection independent of the H5N1 isolate (Fig. 5). In contrast, all mice vaccinated with VSV-EBOV-HAfl or VSV-HAfl were completetly protected aginast lethal challenge (Fig. 5), but mice challenged with A/Indonesia/5/2005 showed minor body weight loss on days 4–6 after challenge (Fig. 5c). Taken together, the VSV vectors expressing HAfl confer H5 cross-protection in the mouse model.

## Discussion

The 1997 outbreak of human H5N1-caused disease in Hong Kong was controlled with the depopulation of poultry.^37^ However, while this outbreak was contained, HPAI H5N1 viruses have been circulating in poultry for almost two decades now and have spread to more than 60 countries.^38^ The broad geographic distribution of HPAI H5N1 viruses and the risk of transmission to humans causing severe pneumonia with high case fatality rates^39^ are a major concern to animal and human health since years. Treatment is an option for individual human cases but if these HPAIs gain transmissibility for humans, vaccines are likely the only public health measure to fight an epidemic or potential pandemic. In this study, we used the well-characterized VSV-EBOV vaccine as our starting platform as it has advantages over other vaccine approaches such as ease of genetic modification, efficient and cost-effective manufacturing, proven human safety and immunogenicity profile, and potential favorable immune cell targeting.^27,28^

To define a more optimized vaccine approach, we generated several different VSV-EBOV-based vaccine vectors and compared the protective efficacy against HPAI H5N1 virus challenge in the mouse model to a VSV-HAfl vector without the EBOV GP. Despite promising results from previous studies in chickens showing that adjuvanted subunit vaccines consisting of the trimeric H5 sHA (sHAzip) induced high levels of cross-neutralizing antibodies (clade 1 and 2.3.4),^34,35^ we could not demonstrate convincing protection with the sHAzip-expressing VSV vectors in this study (Fig. 2, Supplementary Fig. 2C). In fact, VSV-sHA-EBOV without the trimerization sequence performed better than the sHAzip-expressing vectors with complete protection following prime/boost vaccination (Supplementary Fig. 2C). In contrast to all the sHA-based vaccines, single doses of the VSV-EBOV-HAfl or VSV-HAfl vectors were sufficient to provide complete protection from lethal homologous H5N1 challenge in mice (Fig. 2). Thus, in our study, the VSV vectors expressing HAfl are superior over those expressing sHA or sHAzip.

It should be noted that vaccine doses in this study were about 100 to 1000 times lower than those used in previous VSV-based HPAIV H5N1 vaccine studies.^32,36,40^ This is an important observation, as lower-dose vaccination would likely reduce potential adverse effects of vaccination as has been reported ocassionally from human clinical trials using VSV-EBOV vaccination.^27^ Recently, it has been shown that low-dose vaccination with VSV-EBOV does not compromise protective efficacy in nonhuman primates.^41^ Lower-dose vaccination would also have a beneficial effect on vaccine manufacturing.

Currently, H5 HPAI viruses have been classified into several clades based on phylogenetic analysis of their HA genes.^42^ Notably, mainly clade 2 viruses have evolved rapidly and extensively in recent years, and the continued evolution of this particular virus has heightened the concern for a pandemic.^43^ Thus, here we selected eight viruses from clade 2 and one virus from clade 1 (Supplementary Table 1) to investigate the cross-neutralizing nature of the vaccine-induced antibody response by HI test. We found that a prime/boost vaccination with the VSVs expressing HAfl elicited cross-neutralizing antibodies against all tested H5 viruses (Table 1) suggesting that these vaccine vectors will likely cross-protect. The presence of HI antibodies with titers of ~1:40 is considered protective^44^ as demonstrated in previous animal studies using poxvirus-based vaccination.^45^ Cross-protection could indeed be demonstrated in mouse challenge experiments utilizing three different H5 clade 2 isolates (Fig. 5) highlighting the cross-protective potential of the vaccines.

While prime/boost vaccination with VSV-sHAzip-EBOV or VSV-sHA-EBOV induced cross-neutralizing antibodies, the responses were generally lower in titer and detected in fewer animals. Previous studies have shown that the influenza virus HA stem has the potential to induce broad protective immunity^46^ and that the removal of the transmembrane domain may affect the native conformation of the HA stem potentially destroying those conformational antibody epitopes.^47^ Thus, the finding that our VSV vectors expressing sHA/sHAzip did perform worse than those expressing HAfl is likely due to the specific design of expressing a soluble antigen that lacks both the transmembrane and cytoplasmic domains.

Our studies demonstrate that VSV-based vaccines expressing HAfl are superior over those expressing modified sHA. However, this study did not provide any data supporting an advantage of including VSV-EBOV as part of the vector design over just expressing VSV-HAfl as both vectors performed similarly well with no statistically significant difference in efficacy following single-dose or prime/boost administration (Fig. 2) nor in antibody responses (Fig. 3, Table 1). The lack of differences in HA-specific antibody responses is not necessarily in line with higher expression of HAfl following VSV-HAfl infection in tissue culture (Fig. 1b), but replication may be different in vivo. On the other hand, the postulated favorable immune cell targeting through VSV-EBOV^30,48–50^ may balance the advantage of higher antigen expression by VSV-HAfl.

VSV-EBOV has been shown to induce rapid protective immune responses in preclinical and clinical studies.^27,28^ Thus, this platform has the potential to be utilized as an emergency vaccine. While we could not demonstrate a significant difference between the VSV-HAfl and VSV-HAfl-EBOV vaccine in regard to fast-acting properties, protection after immunization on day −1 is marginally better with VSV-EBOV-HAfl than VSV-HAfl (Fig. 4). This difference could be due to the favorable immune cell targeting of the EBOV GP,^30,48–50^ but further studies with bigger animal group sizes are needed to prove this hypothesis. Previous studies demonstrated that VSV-based vaccines provide rapid protection via involvement of the innate immune system combined with an early adaptive response,^26^ suggesting that the VSV-EBOV-HAfl and VSV-HAfl vaccines may induce innate immune responses that are able to control the challenge virus, allow for the adaptive immune system to catch up and lead to protection of the mice. Nevertheless, the fast-acting feature makes this vaccine extremely valuable for the public health response during an epidemic or pandemic as the vaccine could be strategically administered to more vulnerable populations such as elderly and hospitalized people keeping in mind the replicative nature of the vaccine vectors.

VSV-based H5 influenza virus vaccine candidates have advantages compared to the currently used influenza virus vaccines including the ease of generation of the vectors as well as the vaccine production in cell lines which are already approved for manufacturing of human vaccines.^51^ A switch to cell line production would eliminate concerns regarding allergies to egg proteins. The downside of an attenuated, replication-competent vaccine approach such as VSV is adverse reactions to vaccination. However, previous preclinical vaccine work using the VSV platform, including immunization of several immune-compromised animal species, as well as clinical trials with VSV-EBOV demonstrated low levels of vaccine-related adverse effects resulting in the general conclusion that the VSV vaccine platform is safe.^27,28^ In addition, VSV-based replicating vaccines are efficacious at lower doses compared to non-replicating approaches and do not require adjuvants.^52^

In conclusion, we have developed two VSV-based vaccine candidates, VSV-EBOV-HAfl and VSV-HAfl, that provide proof-of-concept for rapid protection against HPAI virus infection that are mediating cross-neutralizing responses. If clinical development confirms the promise of being fast-acting and strongly protective, VSV-based vectors might be a promising approach for the development of a pan-H5 influenza virus emergency vaccine.

## Methods

### Ethics statement

All infectious work was performed at the required containment level at the Integrated Research Facility, Rocky Mountain Laboratories (RML), Division of Intramural Research (DIR), National Institute of Allergy and Infectious Disease (NIAID), National Institutes of Health (NIH). Vaccinations were carried out in BSL2 settings; H5N1s were handled exclusively under maximum containment. The animal work was approved by the Institutional Animal Care and Use Committee (IACUC) and performed according to the guidelines of the Association for Assessment and Accreditation of Laboratory Animal Care, International and the Office of Laboratory Animal Welfare. All procedures on animals were carried out by trained and certified personnel following standard operating procedures (SOPs) approved by the Institutional Biosafety Committee (IBC). Humane endpoint criteria in compliance with IACUC-approved scoring parameters were used to determine when animals should be humanely euthanized.

### Cells and viruses

African green monkey kidney (Vero E6) cells were grown in Dulbecco’s modified Eagle’s medium (DMEM) (Sigma-Aldrich) containing 2% or 10% fetal bovine serum (FBS), 2 mM l-glutamine, 50 U/ml penicillin, and 50 μg/ml streptomycin (all from Thermo Fisher Scientific). Baby hamster kidney (BHK)–T7 cells were grown in minimum essential medium (MEM) (Thermo Fisher Scientific) containing 10% tryptose phosphate broth (Thermo Fisher Scientific), 5% FBS, l-glutamine, penicillin, and streptomycin. Madin-darby canine kidney (MDCK) cells were grown in Eagle’s minimum essential medium (EMEM) containing 10% FBS, l-glutamine, penicillin, streptomycin, MEM non-essential amino acid (Thermo Fisher Scientific), and bicarbonate (Thermo Fisher Scientific). The H5N1 challenge viruses A/Vietnam/1203/2004 (clade 1; kindly provided by Kanta Subbarao, NIAID/NIH, USA), A/Anhui/2005 (clade 2.3.4), A/Duck/Vietnam/2010 (clade 2.3.2.1a), and A/Indonesia/5/2005 (clade 2.1.3.2) were propagated and titered on MDCK cells. The following candidate vaccine viruses were obtained from the Centers for Disease Control and Prevention in Atlanta, USA, and propagated similarly to the H5N1 influenza viruses on MDCK cells: A/Indonesia/5/2005-like IBCDC-RG2, A/India/NIV/2006-like IBCDC-RG7, A/Egypt/N03072/2010-like IDCDC-RG29, A/Hubei/1/2010-like IDCDC-RG30, A/Cambodia/X0810301/2013-like IDCDC-RG34B, A/Guizhou/1/2013-like IDCDC-RG35, A/chicken/Bangladesh/11rs1984-30/2011-like RG36, A/Sichuan/26221/2014-like IDCDC-RG42A, and A/gyrfalcon/Washington/41088-6/2014-like IDCDC-RG43A.

### Plasmid construction and VSV recovery

The H5 HAfl ORF was constructed using the entire HA cDNA sequence of H5N1 A/Vietnam/1203/2004. The soluble HA (sHA) ORF was generated from the HAfl ORF by deleting the transmembrane domain and replacing the sequences encoding the polybasic cleavage site between HA1 and HA2 (PQRERRRKKRG) by one preventing cleavage (PQIETRG). The sHA with leucine zipper (sHAzip) was constructed of the sHA ORF by adding a GCN4pll sequence for trimerization.^35^ All ORFs were cloned into the pATX-VSV-EBOV plasmid encoding the EBOV-Mayinga GP.^53^ Replication-competent recombinant VSVs (VSV-EBOV-sHAzip, VSV-sHAzip-EBOV, VSV-sHA-EBOV, VSV-EBOV-HAfl, and VSV-HAfl) were generated as described previously.^24^ The complete sequence of the VSV vaccines was confirmed by Sanger sequencing. Detailed sequence information can be obtained from the authors upon request. The titer of each virus stock was quantified using standard plaque and TCID_50_ assays on Vero E6 cells. The same vaccine virus stock was used for all in vitro and in vivo work.

### Growth kinetics

Vero E6 cells were grown to confluency in a 12-well plate and infected in triplicate with VSVwt, VSV-EBOV, VSV-EBOV-sHAzip, VSV-sHAzip-EBOV, VSV-sHA-EBOV, VSV-EBOV-HAfl, and VSV-HAfl (MOI of 0.01). The inoculum was removed, cells were washed three times with DMEM, and covered with DMEM containing 2% FBS, 2.5 μg/ml TPCK trypsin (Thermo Fisher Scientific), and 1 U/mg NA from *Vibrio cholerae* (Sigma-Aldrich). TPCK trypsin and NA are required for the propagation of the VSV-HAfl vaccine. Supernatant samples were collected at 0, 24, 48, and 72 h post-infection and stored at −80 °C. The titer of the supernatant samples was determined performing TCID_50_ assay on Vero E6 cells.

### Western blot analysis

Samples were generated in parallel from each vaccine virus stocks produced in Vero E6 cells mixed 1:1 with sodium dodecyl sulfate-polyacrylamide (SDS) gel electrophoresis sample buffer containing 20% β-mercaptoethanol and heated to 99 °C for 10 min. SDS-PAGE with all samples was performed in parallel on TGX criterion pre-cast gels (Bio-Rad Laboratories) (Supplementary Fig. 1). Subsequently, proteins were transferred to a Trans-Blot polyvinylidene difluoride membrane (Bio-Rad Laboratories). The membrane was blocked for 3 h at room temperature in PBS with 3% powdered milk and 0.05% Tween 20 (Thermo Fisher Scientific). Protein detection was performed using the following rabbit or mouse primary antibodies: anti-HA 1:1000 (cat. #11062-T54-100, Sino Biological Inc.), anti-EBOV GP (ZGP 12/1.1, 1 μg/ml; kindly provided by Ayato Takada, Hokkaido University, Sapporo, Japan), and anti-VSV M (23H12, 1:1000; Kerafast Inc.). After horse-raddish peroxidase (HRP)-labeled secondary antibody staining using either anti-mouse IgG (1:10,000) or anti-rabbit IgG (1:5000) (mouse cat. #715-035-151; rabbit cat. #711-035-152; both Jackson ImmunoResearch), the blots were imaged using the SuperSignal West Pico chemiluminescent substrate (Thermo Fisher Scientific) and a FluorChem E system (ProteinSimple).

### Vaccination and protective efficacy in mice

Groups of female Balb/c mice (*n* = 16) were vaccinated IM with 1 × 10^4^ pfu of the VSV-based vectors in 0.1 ml (two sites, 0.05 ml each) on day −42 and −21 (prime/boost vaccination) or −21 only (single-dose vaccination). On the day of challenge (day 0), four animals in each group were euthanized for serum collection. The remaining 12 animals in each group were challenged intranasally (IN) with 100 LD_50_ (400 TCID_50_) of HPAI H5N1 virus A/Vietnam/1203/2004. On day 6 post challenge, four animals in each group were euthanized and samples were collected for serology. The remaining eight mice were monitored until 28 days post challenge when a terminal blood sample was collected prior to euthanasia.

For the time to immunity study, groups (*n* = 8) of female Balb/c mice were IM vaccinated on day −7, −3, or −1 with 1 × 10^4^ pfu of the VSV-HAfl or VSV-EBOV-HAfl vaccine in 0.1 ml (two sites, 0.05 ml each). VSV-EBOV was used as a control. All the groups were challenged IN with 100 LD_50_ (400 TCID_50_) of HPAI H5N1 virus A/Vietnam/1203/2004. Surviving mice were monitored until day 28 post infection.

For the H5 cross-protection study, groups (*n* = 6) of female Balb/c mice were IM vaccinated on day −28 with 1 × 10^4^ pfu of the VSV-HAfl or VSV-EBOV-HAfl vaccine in 0.1 ml (two sites, 0.05 ml each). VSV-EBOV was used as a control. All the groups were challenged IN with 400 TCID_50_ of HPAI H5N1 viruses, namely A/Anhui/2005, A/Duck/Vietnam/2010, and A/Indonesia/5/2005. Surviving mice were monitored until day 28 post infection.

### Enzyme-linked immunosorbent assay

Serum samples from H5N1-infectd mice were inactivated by gamma-irradiation and used in BSL2 according to IBC-approved SOPs. ELISA plates were coated with 1 µg/ml (50 µl/well) of recombinant influenza HA (H5) (A/Vietnam/1203/2004) antigen (IBT Bioservices). After three washes with PBS/Tween, plates were blocked with 1% BSA in PBS for 3 h at room temperature, followed by three additional washes with PBS/Tween. The plates were incubated with fourfold serial dilutions of the mouse serum samples for 1 h at 37 °C, and washed three times with PBS/Tween. Bound antibodies were visualized with horseradish peroxidase-conjugated goat anti-mouse IgG (H+L) (Jackson ImmunoResearch) at a 1:1000 dilution and TMB substrate (KPL). The reaction was measured using the Synergy™ HTX Multi-Mode Microplate Reader (BioTek). Titers were calculated by a 4-parameter curve fitting model using Microsoft Excel software. The cutoff value was set as the mean optical density plus three standard deviations of the control samples.

### HI assay

HI assays were performed using eight hemagglutination units/25 μl of the different H5 viruses incubated with 25 μl of the fourfold serial dilutions of each mouse serum sample (day 0) in round-bottom 96-well plates for 1 h at room temperature. Then 50 μl of a 0.8% turkey red blood cell solution (Innovative Research) was added to each well. Plates were covered and incubated for 30 min on ice. Hemagglutination titers were determined by the reciprocal of the last dilution containing agglutinated turkey red blood cells. HI titers represent the highest serum dilution that completely inhibited hemagglutination.

### Statistical analysis

Statistical analysis was performed in Prism 7 (GraphPad). Data presented in Figs 1c, 2 (upper panels), 4 (upper panels), 5 (left panels), Supplementary Fig. 1C, and Supplementary Fig. 1D (upper panels) were examined using two-way ANOVA with Tukey’s multiple comparison to evaluate statistical significance at all timepoints between all groups. Significant differences in the survival curves shown in Figs. 2 (lower panels), 4 (lower panels), 5 (right panels), and Supplementary Fig. 1D (lower panels) were determined performing Log-Rank analysis. Data presented in Fig. 3 and Supplementary Fig. 2 were analyzed for statistical significance using one-way ANOVA with multiple comparison. Statistical significance is indicated as follows: *p* < 0.0001 (****), *p* < 0.001 (***), *p* < 0.01 (**) and *p* < 0.05 (*).

### Reporting summary

Further information on research design is available in the Nature Research Reporting Summary linked to this article.

## Supplementary information

Supplementary Information

Reporting Summary

## Data Availability

The data supporting the findings of this study are available from the corresponding author upon reasonable request.
