# Supplementary material for: A single dose of a vesicular stomatitis virus-based influenza vaccine confers rapid protection against H5 viruses from different clades
Source: NPJ Vaccines. 2020 Jan 10;5:4. doi: 10.1038/s41541-019-0155-z (PMC6954110; doi:10.1038/s41541-019-0155-z)
Supplement: Supplementary file 1 — Supplementary Information [file 41541_2019_155_MOESM1_ESM.pdf]

A single dose of a vesicular stomatitis virus-based influenza vaccine confers rapid protection against H5 viruses from different clades

Wakako Furuyama, Pierce Reynolds, Elaine Haddock, Kimberly Meade-White, Mai Quynh Le, Yoshihiro Kawaoka, Heinz Feldmann, and Andrea Marzi

Supplementary Figure 1
Supplementary Figure 2
Supplementary Figure 3
Supplementary Table 1

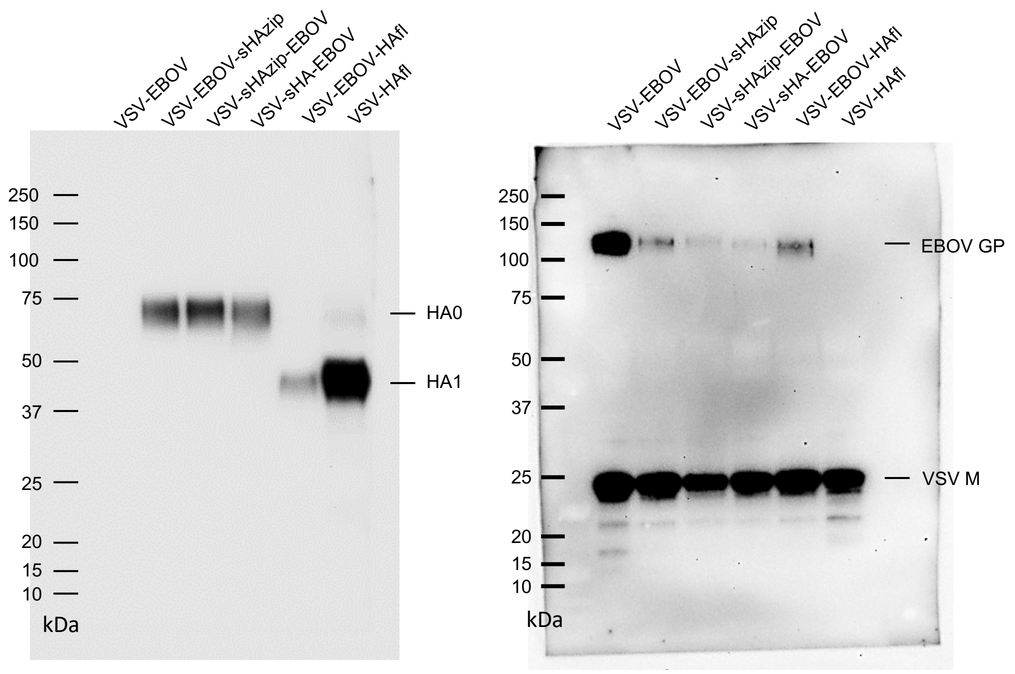

Supplementary Figure 1. Antigen expression. Western blot analysis was performed to confirm antigen expression in the supernatant of VSV-infected Vero E6 cells. HA was detected using a specific antibody to HA (H5) (left panel); EBOV GP and VSV M were detected using mouse monoclonal antibodies specific to each protein (right panel).

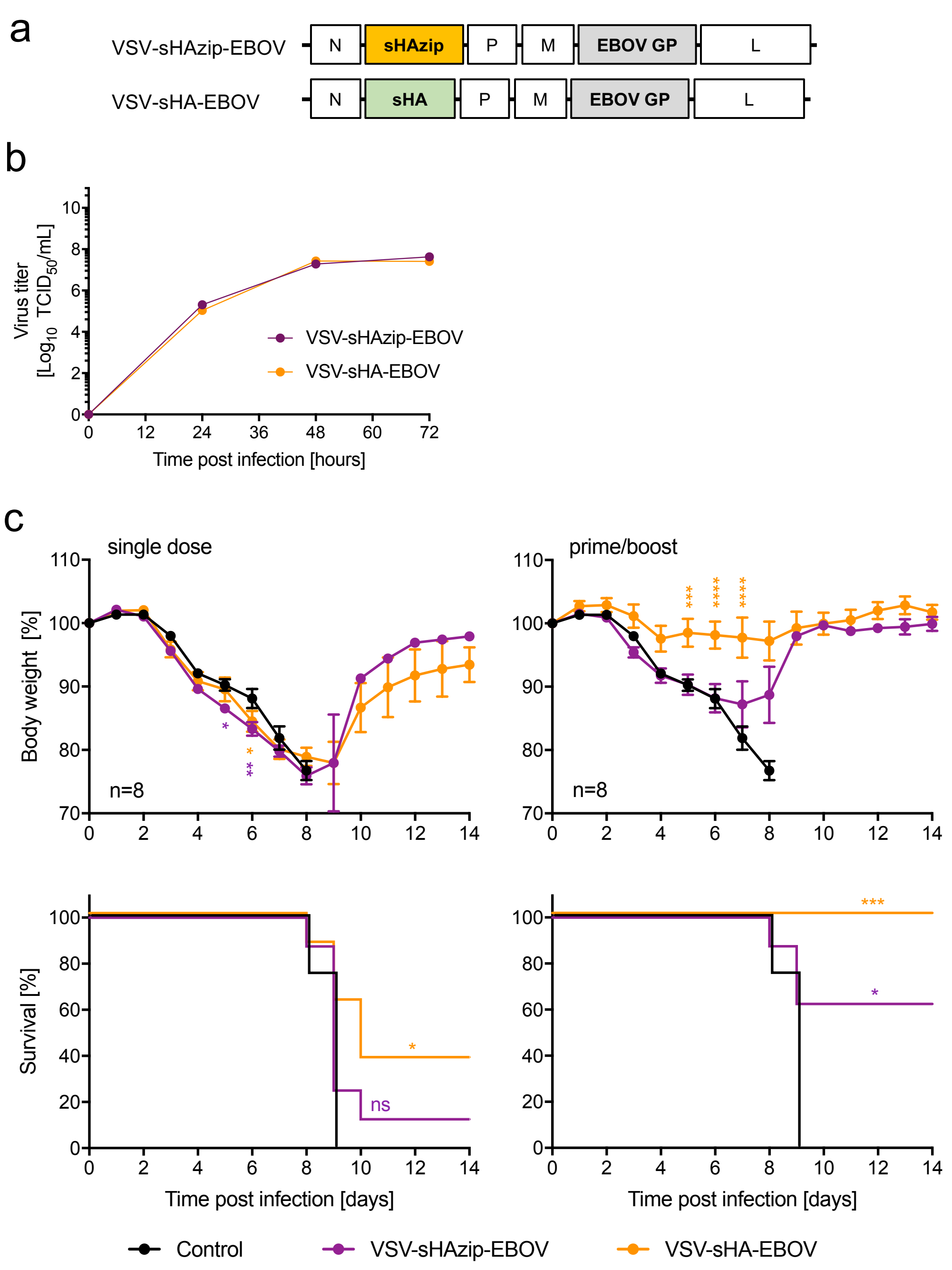

Supplementary Figure 2. Design, *in vitro* characterization, and protective efficacy of the HA-expressing VSV-EBOV vectors. (**A**) Schematic representation of VSV-sHAzip-EBOV and VSV-sHA-EBOV vectors. N, nucleoprotein; P, phosphoprotein; M, matrix protein; EBOV GP, Ebola virus glycoprotein; L, polymerase; sHA, soluble HA; sHAzip, soluble HA protein with zipper sequence. **(B)** *G*rowth kinetics of VSVs propagated on Vero E6 cells. The mean and standard deviation of one experiment performed in triplicates are shown. (**C**) Groups of 8 mice were IM vaccinated with a single dose (left) or a prime/boost (right) of the indicated VSVs on day -21 (left, single dose), or on days -42 and -21 (right, prime/boost) before challenge. On day 0, mice were challenged intranasally with a lethal dose of HPAI H5N1 virus. Body weight (upper panels) and survival curves (lower panels) are shown. Error bars indicate standard error of the mean (SEM). Statistical significance is indicated (p<0.0001 (****), p<0.001 (***), p<0.01 (**) and p<0.05 (*); ns, not significant).

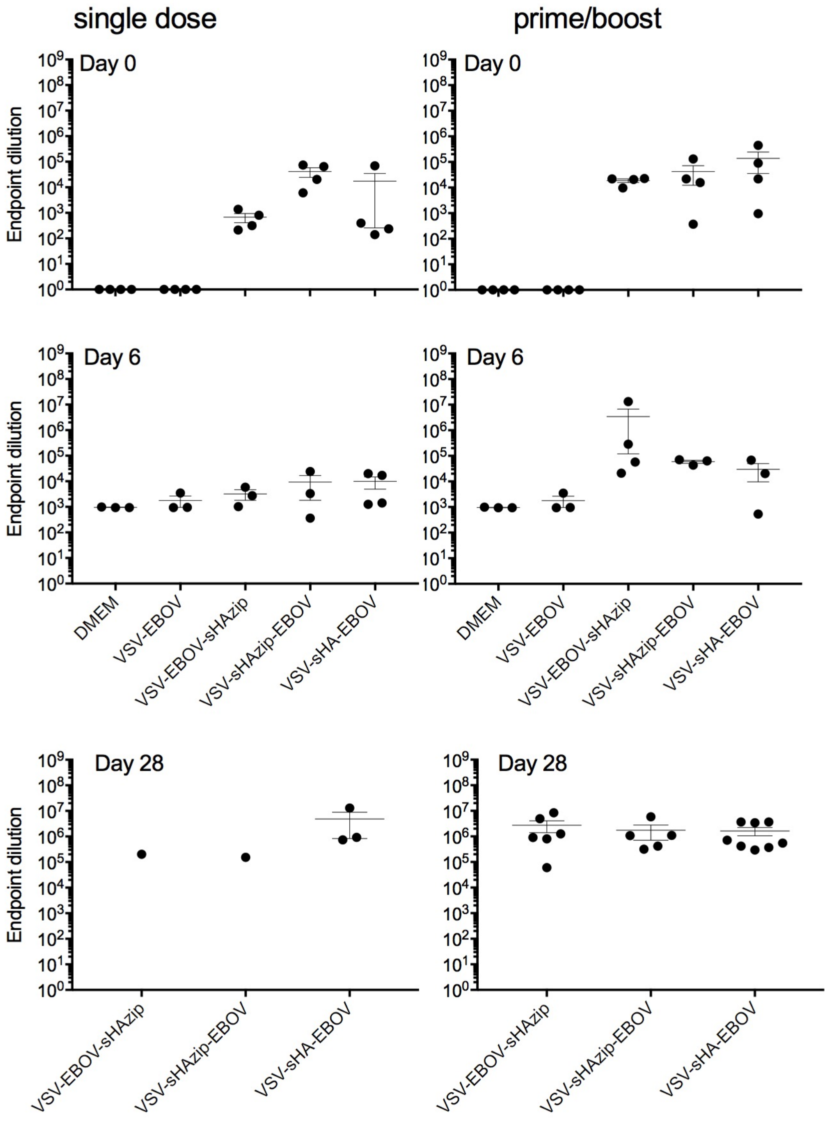

Supplementary Figure 3. Antibody response after VSVs vaccination and H5N1 challenge. Levels of HA (H5)-specific IgG present in the mouse serum samples were analyzed by ELISA. Serum samples from the single dose or prime/boost vaccination with the VSVs were collected on day 0, 6 and 28 post challenge. Error bars indicate standard deviation. Differences in results are not statistically significant.

Supplementary Table 1.
H5 hemaglutinin variants tested for cross-protective potential of the VSV-EBOV-HA-induced antibody response.

Virus names	CDC ID number	HA and NA genes	Clade
IBCDC-RG2	2006716817	A/indonesia/05/2005(H5N1)	2.1
IBCDC-RG7	2007730304	A/India/NIV/2006(H5N1)	2.2
IDCDC-RG29	2011815423	A/Egypt/N03072/2010(H5N1)	2.2.1
IDCDC-RG30	2011815424	A/Hubei/1/2010(H5N1)	2.3.2.1
IDCDC-RG34B	2014755555	A/Cambodia/X0810301/2013(H5N1)	1.1.1
IDCDC-RG35	2014755556	A/Guizhou/1/2013(H5N1)	2.3.4.2
IDCDC-RG36	2014755557	A/chicken/Bangladesh/11rs1984-30/2011(H5N1)	2.3.4.2
IDCDC-RG42A	3000095680	A/Sichuan/26221/2014(H5N6)	2.3.4.4
IDCDC-RG43A	3000095679	A/gyrfalcon/Washington/41088-6/2014(H5N8)	2.3.4.4
